# The National Health Policy for people with disabilities in Brazil: an analysis of the content, context and the performance of social actors

**DOI:** 10.1093/heapol/czac051

**Published:** 2022-06-30

**Authors:** Tereza Maciel Lyra, Maria Socorro Veloso de Albuquerque, Raquel Santos de Oliveira, Gabriella Morais Duarte Miranda, Márcia Andréa de Oliveira, Maria Eduarda Carvalho, Helena Fernandes Santos, Loveday Penn-Kekana, Hannah Kuper

**Affiliations:** Aggeu Magalhães Institute, FIOCRUZ/PE, Av. Professor Moraes Rego, s/n—Campus da UFPE, Cidade Universitária, Recife, PE 50.740-465, Brazil; Center for Medical Sciences, Federal University of Pernambuco, Av. da Engenharia, s/n, Bloco ‘D’, 1º Andar—Cidade Universitária, Recife, PE 50.740-600, Brazil; Center for Medical Sciences, Federal University of Pernambuco, Av. da Engenharia, s/n, Bloco ‘D’, 1º Andar—Cidade Universitária, Recife, PE 50.740-600, Brazil; Center for Medical Sciences, Federal University of Pernambuco, Av. da Engenharia, s/n, Bloco ‘D’, 1º Andar—Cidade Universitária, Recife, PE 50.740-600, Brazil; Medical Sciences College, University of Pernambuco, Av. Gov. Agamenon Magalhães—Santo Amaro, Recife, PE 50.100-010, Brazil; Aggeu Magalhães Institute, FIOCRUZ/PE, Av. Professor Moraes Rego, s/n—Campus da UFPE, Cidade Universitária, Recife, PE 50.740-465, Brazil; Center for Philosophy and Human Sciences, Federal University of Pernambuco, Av. Prof. Moraes Rego, 1235—Cidade Universitária, Recife, PE 50670-901, Brazil; Epidemiology and Public Health, Maternal and Neonatal Health Group, London School of Hygiene & Tropical Medicine, Keppel Street, London WC1E 7HT, UK; International Centre for Evidence in Disability, London School of Hygiene & Tropical Medicine, Keppel Street, London WC1E 7HT, UK

**Keywords:** Disability, policy, Brazil

## Abstract

The purpose of this article is to analyse the circumstances in which the National Health Policy for Persons with Disabilities (PNSPCD) came into place in 2002 and the factors supporting or impeding its implementation from 2002 to 2018. The analysis was based on the Comprehensive Policy Analysis Model proposed by Walt and Gilson and focussed on understanding the context, process, content and actors involved in the formulation and implementation of the Policy. Data were obtained from two sources: document analysis of the key relevant documents and seven key informant interviews. Content analysis was undertaken using the Condensation of Meanings technique. The research demonstrates that the development and implementation of PNSPCD is marked by advances and retreats, determined, above all, by national and international macro-political decisions. The policy was formulated during Fernando Henrique’s governments, under pressure from social movements and the international agenda and constituted a breakthrough for the rights of persons with disabilities. However, progress on implementation only took place under subsequent centre-left governments with the establishment of a care network for people with disabilities and a defined specific budget. These developments resulted from the mobilization of social movements, the ratification of the United Nations Convention on the rights of people with disabilities and the adherence of these governments to the human rights agenda. The coming to power of ultra-right governments triggered fiscal austerity, a setback in the implementation of the care network and a weakening in the content of various social policies related to the care of people with disabilities. During this era, the political approach changed, with the attempt to evade the role of the State, and the perspective of guaranteeing social rights. Undoubtedly, the neoliberal offensive on social policies, especially the Unified Health System, is the main obstacle to the effective implementation of the PNPCD in Brazil.

Key messages

- In Brazil, support for people with disabilities traditionally was through philanthropy and focussed on providing social assistance. Lobbying by people with disabilities led to attention on disability in the health sector, culminating in the formulation of the Policy on Health Care for Persons with Disabilities (PNSPCD) in 2002.
- The implementation of the PNSPCD was strongly affected by different political, economic and social contexts, with moments of advances and setbacks.
- The Brazilian Health System (Sistema Único de Saúde—SUS) and the PNSPCD are marked by chronic underfunding, with some improvements during centre-left governments.
- The rise of an ultra-right and ultra-neoliberal government has negatively influenced social policies, including the PNSPCD by restricting social participation; reducing the scope and content of various policies relevant to the care for people with disabilities and by the defunding process.
- These study results may support the fight for the rights of the movements of people with disabilities and help the consolidation process of the policy of care for people with disabilities in the health system in Brazil.

## Introduction

Globally, there are approximately one billion people with disabilities, with about 200 million experiencing severe functional difficulties (WHO, 2011). This number is likely to increase, given population growth and ageing. In Brazil, according to the 2010 Demographic Census, almost 46 million Brazilians, about 24% of the population, declared that they have some kind of disability, of whom 12.5 million (6.7% of the population) have ‘great’ or ‘total’ difficulty (IBGE, 2010). On average, people with disabilities have greater healthcare needs, yet face widespread difficulties in accessing services (WHO, 2011; UNDESA, 2018; Albuquerque *et al.*, 2019; Kuper and Heydt, 2019). Consequently, disabled people experience worse health and have higher mortality rates, including from coronavirus disease-19 (COVID-19) (WHO, 2011; UNDESA, 2018; Albuquerque *et al.*, 2019; Kuper and Heydt, 2019; Bosworth *et al.*, 2021). Health systems, therefore, need to adapt to better serve the needs of people with disabilities, and this process will be guided by the policy framework.

Brazil has a progressive policy context which should support the inclusion of people with disabilities in the health system. The National Health Policy for people with disabilities (Política Nacional de Saúde da Pessoa com Deficiência—PNSPCD), which is commended by disability rights campaigners around the world, was established in 2002. PNSPCD focusses on six areas: promotion of quality of life of people with disabilities; inclusion in health of people with disabilities; prevention of disability; expansion and strengthening of information mechanisms; organization and operation of care services to people with disabilities and training of human resources. The scope of PNSPCD is ambitious and far-reaching and is consistent with removing barriers identified through the social model of understanding of disability. It calls for the coordination of activities across different governmental and non-governmental sectors to encourage inclusion, protect health and promote effective participation in society for people with disabilities. The ambitions of PNSPCD were reinforced by subsequent laws and policies in Brazil, such as the ‘Living without limits’ plan of 2011. Brazil was also an early signatory to the Convention on the Rights of Persons with Disabilities created in 2006 by the United Nations (UN, 2006). In parallel, the right to healthcare for people with disabilities was supported by the implementation of the Brazilian Unified Health System (Sistema Único de Saúde—SUS). SUS was created in the 1988 Brazilian Constitution after an intense struggle between those arguing that health must constitute a right of citizenship and a duty of the study (e.g. Brazilian Sanitary Reform Movement) and factions promoting the expansion of a liberal private-sector system centred on the commercialization of health (Souto and de Oliveira, 2016). SUS is guided by the principles of universality, integrality and equity (Castro *et al.*, 2019). Its structure is based upon systems in countries like the UK, Sweden, Spain or Canada, and access is guaranteed at no cost to citizens (Marques and Mendes, 2012). However, despite this favourable policy context, there is strong evidence that people with disabilities, still struggle with access to health services in Brazil (Malta *et al.*, 2016; Bright and Kuper, 2018; Sakellariou *et al.*, 2020; Pinto *et al.*, 2021).

In this paper we argue that the PNSPCD, just like the public health system which was meant to implement the policy, had impressive aims of universality, integrality and equity (Paim *et al.*, 2011). However, these goals have not been achieved for the most part because of the neoliberal context and the fiscal austerity that has shaped the national government’s approach to health services as a whole in Brazil, in particular since 2015 (Albuquerque *et al.*, 2019). We argue that while it is important to have a progressive policy, it means little if the context in which it is implemented and the key people involved in the policy implementation process are not truly committed to funding the services needed to make the aspirations of the policy a reality.

Using the Walt and Gilson framework, this article aims to analyse the environment in which the PNSPCD came into place and its implementation from 2002 to 2018, exploring the content of the policy, the context in which the policy was developed, the political process involving the policy and the actions of key actors (Walt and Gilson, 1994). This framework was selected in recognition that the interrelationship between the four categories is important. The actors influence and are influenced by the context in which they live and act. The context is affected by procedural aspects; the political process is affected and determined by the actors involved and the content of the policy will reflect the set of aspects listed above.

Key actors of the PNSPCD transcend the health sector and also include the Social Civil society movements, which are prominent in Brazil. They generally take a person-centred and rights-based approach and focus on provision of support and services, and advocating for the rights of persons with disabilities. For instance, they were early adopters of the terms ‘disability’ and ‘person with disabilities’ as forms of expression, indicating, correctly, that disability is an attribute that distinguishes people, but does not make them inferior, in line with the 2006 International Convention for the Protection and Promotion of the Rights and Dignity of Persons with Disabilities (Sassaki, 2003; Borges and Pereira, 2016; Mello, 2016). This view reinforces the understanding that the social model should guide public policies (Diniz *et al.*, 2007). However, these are shaped in a permanent arena of dispute, in which the political conceptions of those who define them play a key role (Walt and Gilson, 1994). The disability-related civil society movements frequently address specific conditions (e.g. Movimento Downs) and so vary in focus and aims. There is also not always consensus between different civil society movements on the configuration of disability-related policies in Brazil. For instance, some groups and institutions promote the education of children with disabilities in the regular school system, under the principle ‘nothing about us, without us’. Meanwhile, other institutions call for special education, maintained with public resources (Amorim *et al.*, 2019).

The purpose of this article is to analyse the circumstances in which the National Health Policy for Persons with Disabilities (PNSPCD) came into place and factors supporting or impeding its implementation from 2002 to 2018. This analysis of PNSPCD is important as there have been no studies on the process of policy formulation and implementation that have explored the macro aspects and the political, economic and social micro context in which this policy was established; the political process that combined the influence of various national and international social actors and the impacts of adherence to neoliberalism by governments on the implementation and effective financing of the policy in Brazil.

## Materials and methods

The development and implementation of PNSPCD was analysed based on the Comprehensive Policy Analysis Model proposed by Walt and Gilson (Walt and Gilson, 1994) and categorized by Araújo Júnior and Maciel Filho (de Araujo and Filho, 2001) and the Policy Cycle model (Table 1) (Howlett *et al.*, 2009). The Walt and Gilson framework was selected as it is grounded in a political economy perspective and focuses understanding the context, process, content and actors involved in the formulation and implementation of the policy. Alternative approaches and frameworks were considered for the policy analysis, but appeared less relevant as they focussed on specific components, such as describing the process of implementation or networks of stakeholders (Walt *et al.*, 2008). Moreover, the Walt and Gilson framework has been widely implemented, including in low and middle-income settings such as Brazil, allowing comparison with other case studies (O’Brien *et al.*, 2020).

**Table 1.** Policy analysis categories and subcategories

Category	Description	Sub-category	Objectives
Context ^(^^a^^)^	Systemic factors: social, economic, political, cultural, and other environmental conditions	Macrocontext^c^	Describe political, economic and social aspects, focused on society as a whole.
		Microcontext^c^	Describe political, economic and social aspects related to the sector under analysis.
Policy Process/Cycle^(^^a^^) (^^b^^)^	Way in which policies are initiated, developed or formulated, negotiated, communicated, implemented and evaluated	Entry into the political agenda Policy formulation Decision making process Implementation	Understand who influences the entry of a problem on the agenda; how the formulation and decision process for implementing a given policy took place; how such policy was implemented; what factors influenced its content.
Content ^(^^a^^)^	Policy objectives, operational policies, legislation, regulations, guidelines, etc.		Describe the details of the policy under analysis.
Actors ^(^^a^^)^	Influential individuals, groups and organizations. Different actors can participate at each point in the policy cycle.	State Non-state (civil society)	Identify the actors involved, whether individual or collective, their influences, supports or restrictions.

From: ^a^Walt and Gilson (Walt and Gilson, 1994); ^b^Howlet, Ramesh and Pearl (Howlett *et al.*, 2009); ^c^Araújo Júnior; Maciel Filho (de Araujo and Filho, 2001).

Data were obtained from two sources for this analysis: Document analysis and key informant interviews. We undertook a content analysis of the key relevant documents related to the formulation and implementation of the PNSPCD (Table 2). A content analysis was carried out on those key documents. As highlighted by Dalglish, Khalid and McMahon (Dalglish *et al.*, 2021), policy documents were reviewed to describe the content or categorize the approaches to the health policies analysed in this article.

**Table 2.** Main documents analysed

Document	Main content
**Law/Policy**	**Specification on access to healthcare for people with disabilities.**
Federal Constitution Brazil. 1988. Constituição da República Federativa do Brasil. Brasília (DF): Supremo Tribunal Federal; Secretaria de Altos Estudos; Pesquisas e Gestão da Informação. http://www.stf.jus.br/arquivo/cms/publicacaoLegislacaoAnotada/anexo/CF.pdf, accessed 5 August 2021.	It is the common competence of the Union, the States, the Federal District, and the Municipalities to take care of public health, assistance, and the protection of people with disabilities.
PNSPCD/Ministerial Ordinance 1060 Brazil. 2002. Portaria Ministerial N° 1060. Brasília (DF): Ministério da Saúde. https://bvsms.saude.gov.br/bvs/saudelegis/gm/2002/prt1060_05_06_2002.html, accessed 5 August 2021.	Rehabilitation of the Disabled Person, the protection of their health and the prevention of diseases that determine the appearance of disabilities, through the development of articulated actions among the various sectors and the effective participation of society.
Living Without Limits Plan/ Decree 7612 Brazil. 2011. Decreto N° 7612. Brasília (DF): Presidência da República; Casa Civil; Subchefia para Assuntos Jurídicos. http://www.planalto.gov.br/ccivil_03/_ato2011-2014/2011/decreto/d7612.htm, accessed 5 August 2021.	Promote the access to education, health care, social inclusion, and the accessibility for the disabled person.
Care Network for People with Disabilities within the SUS/ Ministerial Ordinance 793 and Ministerial Ordinance 835 Brazil. 2012. Portaria Nº 835. Brasília (DF): Ministério da Saúde; Gabinete do Ministro. https://bvsms.saude.gov.br/bvs/saudelegis/gm/2012/prt0835_25_04_2012.html, accessed 5 August 2021. Brazil. 2012. Portaria Nº 793. Brasília (DF): Ministério da Saúde; Gabinete do Ministro. https://bvsms.saude.gov.br/bvs/saudelegis/gm/2012/prt0793_24_04_2012.html, accessed 5 August 2021	The Care Network for the Disabled Person is organized in: I—Primary care; II- Specialized Attention in Hearing, Physical, Intellectual and Visual Rehabilitation, Ostomy and in Multiple Deficiencies; III- Hospital care and emergency service. Institutes financial incentives for the construction, renovation or expansion of physical location and orthopaedic workshop service, as well as for the acquisition of equipment and other permanent materials.
Brazilian Inclusion Law/ Law 13 146 Brazil. 2015. Lei Nº 13.146. Brasília (DF): Presidência da República; Secretaria-Geral; Subchefia para Assuntos Jurídicos. http://www.planalto.gov.br/ccivil_03/_ato2015-2018/2015/lei/l13146.htm, accessed 5 August 2021.	Comprehensive health care for people with disabilities is ensured at all levels of complexity, through the Unified Health System, guaranteeing universal and equal access.
PNAB/ Ministerial Ordinance 2436 Brazil. 2017. Portaria Nº 2436. Brasília (DF): Ministério da Saúde; Gabinete do Ministro. https://bvsms.saude.gov.br/bvs/saudelegis/gm/2017/prt2436_22_09_2017.html, accessed 5 August 2021.	Ensure adequate infrastructure and good conditions for the operation of Basic Health Units, guaranteeing space, furniture, and equipment, as well as accessibility for people with disabilities, in accordance with current regulations.
Decree 9759 Brazil. 2019. Decreto N° 9759. Brasília (DF): Presidência da República; Secretaria-Geral; Subchefia para Assuntos Jurídicos. http://www.planalto.gov.br/ccivil_03/_ato2019-2022/2019/decreto/D9759.htm, accessed 5 August 2021.	Extinguishes the National Social Participation Policy (Decree Nº 8.243/2014) which aimed to strengthen and articulate the mechanisms and democratic instances of dialogue and joint action between the federal public administration and civil society.
PREVINE/ Ministerial Ordinance 2979 Brazil. 2019. Portaria Nº 2979. Brasília (DF): Ministério da Saúde; Gabinete do Ministro. https://www.in.gov.br/en/web/dou/-/portaria-n-2.979-de-12-de-novembro-de-2019-227652180, accessed 5 August 2021.	Institutes the PREVINE Brazil Program, which establishes a new funding model for the cost of Primary Health Care within the scope of the Unified Health System.
Decree 10 502 Brazil. 2020. Decreto Nº 10.502. Brasília (DF): Presidência da República; Secretaria-Geral; Subchefia para Assuntos Jurídicos. http://www.planalto.gov.br/ccivil_03/_ato2019-2022/2020/decreto/D10502.htm, accessed 5 August 2021.	Establishes the National Policy on Special Education. With the objective of implementing programs and actions aimed at guaranteeing the rights to education and specialized educational services for students with disabilities, pervasive developmental disorders and high abilities or giftedness.

Key informant interviews were undertaken to explore the context, policy process and actors relevant to the formulation and implementation of the PNSPCD. We developed an interview guide to explore the following aspects: the political, economic and social context of Brazil during the relevant time period; the context of the SUS in the process of policy formulation and implementation; the inclusion of the needs of people with disabilities in the policy process; identification of key social actors in the political process of agenda building, formulation and implementation of the policy. Seven key informants were interviewed, including: (1) former health minister; (2) former policy manager; (3) representative of The National Organization of the Blind (Professor and researcher at a Public University, former Executive Secretary of the Health National Council); (4) professor of disability research; (5) former National Manager of the Health Care Policy of the PNSPCD; (6) leading activist campaigning for the rights of people with disabilities and member of the National Health Council; (7) member of the Ministry of Health of the technical team of the current National Policy Coordination in 2019. Three of the key informants were people with disabilities.

Interviews took 1–2 h and were conducted by pairs of researchers and were recorded and transcribed. The interviews were read by two researchers, independently, and then meetings were held for agreement of the main themes and categories. The interviews were analysed following the content analysis model (Bardin, 1993), opting for the content analysis technique called Condensation of Meanings, proposed by Kvale (Kvale, 1996).

All interviews were conducted, transcribed and analysed by a team of researchers in Brazil, all with a PhD degree completed or in progress, with extensive experience in qualitative research.

The research was submitted and approved by the Ethics Committees of the authors’ institutions. All ethical precepts were followed, and the interviewees’ confidentiality was assured.

## Results and discussion

**Figure 1.**
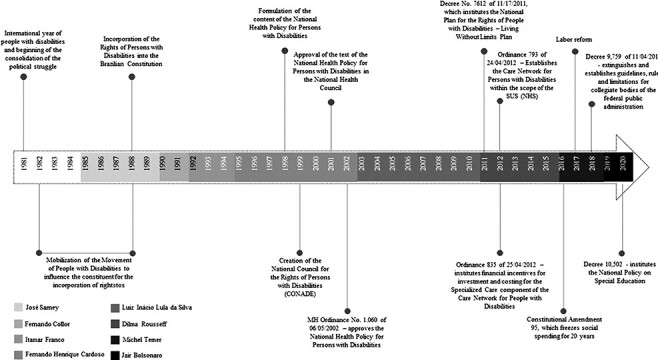
Policy development timeline

The PNSPCD was enacted in 2002, and so was influenced by the following governmental periods in Brazil: FHC—Fernando Henrique Cardoso (Brazil Social Democracy Party, 1995–2002); Lula and Dilma (Worker’s Party, 2003–15); Temer (Brazilian Democratic Movement, 2015–18) and Bolsonaro (Social Liberal Party, now without party, term 2019–22). These terms relate to three major political-ideological periods which influenced the formulation and implementation of the PNSPCD: the first (FHC) marked by the entry of the neoliberal agenda in the country; the second (Lula/Dilma) of neo-developmentalist model, which sought the economic growth of Brazilian capitalism through neoliberal economics but with the inclusion, via social public policies, of an excluded portion of society (Boito *et al.*, 2014) and the third (Temer/Bolsonaro) of an ultraliberal political platform with setbacks in the social agenda (Figure 1). This paper considers the processes of (dis)continuities, advances and setbacks of implementation of the PNSPCD in each of these socio-historical moments, focussing on both the macro and the micro context. The timeline summarized in Figure 1.

This chronological division was designed in an attempt to demarcate the main developments in politics and economics and their repercussions on this sectoral policy. However, several aspects of the previous governmental projects permeate and often intensify in subsequent governments. The main actors are summarized in Table 3.

**Table 3.** Summary of actors and their characteristics in relation to the development and implementation of PNSPCD

	Characteristics			
Actors	Role/Performance	Contribution to the PNSPCD	Supporting factors for PNSPCD implementation	Detracting factors for PNSPCD implementation
United Nations	Established the Convention on the Rights of Persons with Disabilities (UNCRPD) reiterating the right to health and rehabilitation for persons with disabilities.	Ratification of the Convention on the Rights of Persons with Disabilities in Brazil in 2008 supported the ambitions of PNSPCD.	High level political commitment indicated through ratification. Periodic independent monitoring of Brazil’s implementation of UNCRPD, including with respect to health.	The commitments made in UNCRPD are not enforced.
Civil society	Advocates for the rights of persons with disabilities	Participated in the formulation of the PNSPCD and continued mobilization	Active, mobilized and committed supporters.	Civil society groups are fragmented.
Philanthropic sector	Providers of (rehabilitation) services	Demonstrated the need and ability to provide rehabilitation services.	Participated in the provision of services aimed at people with disabilities, with resources from the public fund.	Use of public funds for the philanthropic sector was detrimental to the development of these services within SUS.
Fernando Henrique Cardoso	Former President (1995/2002)	Oversaw the establishment of PNSPCD	Intense mobilization of civil society, including people with disabilities, for the guarantee of rights, and the formulation of the PNSPCD.	Neoliberal political and economic context, generally adverse to a focus on disability-inclusive health, including chronic underfunding of SUS.
Lula and Dilma	Former Presidents (2003/2014)	Oversaw establishment of additional plans to support PNSPCD implementation (e.g. Network of Care for Persons with Disabilities, Living without Limits plan) including allocation of financial resources.	Centre-left political agenda with a focus on social inclusion. Ratification of UNCRPD and guarantee of political resources for social movements.	Continued underfunding and inequalities in SUS. Insufficient funding for implementation of PNSPCD and fragmentation of services.
Temer and Bolsonaro	Former president and current president (2014 to date—2021)	Supported substantial regression of achievements through reducing funds available to SUS and related institutions and dismantling legal frameworks	Resistance by civil society and others to dismantling of disability provisions.	Economic recession led to fiscal adjustment and cuts to SUS. Ultra-liberalisation and reduced focus on public provision of services.

### Fernando Henrique Cardoso (1995–2002) and prior period: the context of policy formulation

The era prior to FHC was characterized by a neoliberal political and economic context, generally adverse to a focus on disability-inclusive health. By prioritizing economic measures that reduced social spending, the State moved away from its role as a promoter of social well-being and took on the role of manager of the free movement of capital (Sa, 2016). However, in this earlier era, there were key influences that supported the eventual development of the PNSPC. First, the rights of people with disabilities were recognized in the 1988 Federal Constitution (FC). Second, the right to health for all Brazilian citizens specified in the Constitution was supported with the creation of the Brazilian Unified Health System (SUS) in 1988. And prior to this, in 1981, the proclamation by the United Nations of the International Year of Disabled Persons helped to place disability on the public agenda.

*“[*...*] I think that the important thing was the fact that we have the UN international year, and it has been in Brazil a pro-movement force, that the social movements have organized themselves even during the period of the military regime, that we had the possibility to intervene, and we are the only popular amendment that entered the Federal Constitution in ‘88* (Person with disability, former policy manager).

The FHC period was characterized by a centre-right government that adopted the neoliberal economic policy—implemented in Brazil since the previous government and widely expanded in the FHC governments. This government agenda prioritized (1) trade opening; (2) financial liberalization; (3) liberalization of the foreign investment regime; (4) privatizations of public services and state-owned companies; (5) labour market deregulation and the encouragement of the practice of outsourcing (Homedes and Ugalde, 2005; Massimo, 2013). In addition to these priorities, public resources to finance social policies were reduced in order to promote the payment of public debts (Oliveira, 2010).

The social and health experiences of this period, analysed by Castro, (Castro, 2020) were characterized by social exclusion. Overall, 60% of the Brazilian population work in the informal sector and social inequality was very high, with the richest 10% receiving half of the income and 35% of the population living below the poverty line. Health outcomes were also poor, for instance, the infant mortality rate was 23.4 per thousand live births. The establishment of SUS in 1990 was an important step to addressing these issues, but it has been plagued by underfunding and geographic inequalities from its inception which reduced its potential for impact and equalization of health access and outcomes. (Castro *et al.*, 2019)

With regard to health actions aimed at people with disabilities, the Brazilian experience was marked by the Poliomyelitis epidemic in the 1950s. The main response was carried out by philanthropic institutions hired by the Brazilian Legion of Social Assistance. According to one of the interviewees, this philanthropy-driven response has had a lasting influence on the organization of health services for people with physical disabilities. In subsequent decades, this philanthropy-driven response has expanded to other areas, incorporating services for people with other types of disabilities. The positive result of the introduction of these initiatives was that it brought other social actors such as health professionals, managers of rehabilitation services and professionals from Non-Governmental Organizations (NGOs) to the political process of struggle for improvements in health services (Maior, 2017).

One positive move during this era was that the political movement composed of people with disabilities, although not homogeneous, increasingly joined efforts in the wake of the return to democracy. They formed new organizations, advocated nationally for strategies to fight for equal opportunities and the fulfilment of rights (Lanna Junior, 2010). Therefore, the organization of the movement of people with disabilities which lobbied for inclusion and the right to health precedes the enactment of the PNSPCD in 2002 and was a key player in making it happen.

Despite this generally unfavourable political and economic context, the guidelines that make up the content of the PNSPCD should be considered a milestone for supporting the needs of people with disabilities in healthcare and the achievement of advocacy groups of people with disabilities, as highlighted by most interviewees. This is because its six guidelines—promotion of quality of life of people with disabilities; inclusion in health of people with disabilities; prevention of disability; expansion and strengthening of information mechanisms; organization and operation of care services to people with disabilities and training of human resources—are ambitious and far-reaching and are consistent with removing barriers identified through the social model of understanding of disability (Saúde, 2002) (Table 2).

However, despite the ambitious scope of the PNSPCD, the influence on the government agenda of the needs of people with disabilities happened incrementally, with slow impact on the formulation of policy, continued centralization of technical knowledge and low political power for its implementation.

*At the time of the formulation of this policy, if on one hand there was a governmental action upon this, but on the other hand the segment was not properly heard. [*...*] it must have gone through the National Health Council (CNS)* (Person with disability and representative of the National Organization of the Blind).

The statements of most interviewees reveal the non-prioritization of the PNSPCD as the formulation process lasted 5 years and with little participation of entities representing people with disabilities. In addition, the transfer of responsibility for the provision of services from the public sector to the philanthropic sector, financed through social resources continued, reaffirming one of the main characteristics of health care for people with disabilities prior to the creation of the PNSPCD. As one interviewees points out, there are places where there is no public policy, but there is always an NGO or association present. The logic of the transfer of public funds to the third sector, which ‘emerges as a new modality aimed at the social function of management and provision of health services, linked to the model of public-private partnerships’ (Morais *et al.*, 2018), represented one of the main proposals of the State Reform in the FHC period.

Finally, it is noteworthy that the absence of funding in the initial period of the creation of the PNSPCD—and in subsequent government periods as will be shown—was a major impediment to its implementation, as highlighted by the interviewees. Consequently, some civil society organizations for people with disabilities got involved in negotiating parliamentary amendments in the national congress.

On the one hand, therefore, the enactment of the PNSPCD in this neoliberal context was a historical moment and achievement of people with disabilities. On the other hand, the neoliberal context resulted in fragmented responses from the Brazilian state and a lack of effective implementation.

### Lula and Dilma (2003–14): advances and contradictions in the policy implementation process

The subsequent governmental era was of the workers’ Party (PT) governments represented by Lula and Dilma (2003–14). This social and historical period signified the entry of a centre-left agenda supported by the dictates of the new-developmentalist policy, which sought to respond to social demands through a set of public interventions. The new-developmentalist agenda adopts a discourse of overcoming neoliberal guidelines.

The political-economic project of this period has been described by Castro as an ‘interstice of social inclusion’, characterized by real growth in employment (39%), a continued downward trend in unemployment (to 7.5%) and a reduction in the population below the poverty line (to 8.1%) (Castro, 2020). The infant mortality rate almost halved, to a national average of 12.4 per 1000 live births in 2014. Another commentator, Pochmann argues that the recovery of the role of the State with the Lula and Dilma governments was essential for these positive impacts, such as a doubling of economic growth in the 1990s, income redistribution particularly for the poor, and the decisive consolidation of social spending with new economic dynamics (Pochmann, 2013). In reality, this period did not break entirely with the neoliberal ideology of the FHC era, including the continued chronic underfunding of SUS (Boito *et al.*, 2014; Carcanholo, 2009).

This period (2003–14) was more favourable for the implementation of the PNSPCD, in terms of both the political and economic environment, both internationally and in Brazil. Notably, the UN adopted the UN Convention on the Rights of Persons with Disabilities in 2006, and it was ratified in Brazil in 2008. Interviewees noted that this provided an important stimulus for the uptake of disability-inclusive policies in Brazil.

*In the first decade of the 21st century we have the approval of the Convention on the Rights of Persons with Disabilities, approved by the UN in 2006, in 2009 the Lula government incorporated the Convention to the Brazilian internal regulatory framework, reproducing the same content of the Convention in Decree 6949 of 2009, and then, finally, we had, after 15 years of discussion in Congress, the law 13.146, which is the Brazilian law of inclusion* (Person with disability, former government policy manager).

Another important milestone in this period was the political decision to establish the Network of Care for Persons with Disabilities within the scope of the SUS, through Ordinance 798/2012. This aimed to expand and specify the requirements of the healthcare system with respect to people with disabilities. For instance, following this legislative framework, the National Government began to fund the Rehabilitation Centres (CER) and defined the responsibilities of each component of the healthcare system for people with disabilities. As highlighted by interviewees, the provision of financial resources was very important, as they enabled strategic actions.

In the two terms of President Lula, the policy maintained the existing services, mainly serving only one type of disability and without investing in the expansion of a comprehensive network of services for different disabilities. In the government of President Dilma, a proposal for an intersectoral plan was developed—the Living without Limits Plan (Table 2). It promoted the development of a network of care for people with disabilities, with the creation of multidisciplinary services that catered to a broad range of impairment types (e.g. physical, sensory). As highlighted by the interviewees, the Plan also enabled the expansion of the network to other regions of the country, beyond the south and southeast, where they were historically concentrated. The importance of this Plan was highlighted by the former minister of health interviewed: *‘[…]Living without limits was a strong and intersectoral agenda that involved several Ministries’.*

Despite these good intentions and plans, the interviewees highlighted that there was a failure to comply with the objectives proposed by the comprehensive health care guidelines and expand comprehensive rehabilitation coverage even after the institution of the Care Network for People with Disabilities.

*[*...*] there was no progress in the integrality of care to the health of people with disabilities. The attention remains fragmented. There has to be a lot of progress in the integration of services. The people with disabilities ends up being a platform for managers, but it is not a priority. There is a lack of resources. We started to make fragmented policies, right, visual rehabilitation, hearing; And then, in 2012, with the launch of Ordinance 793, we included the issue of the network* (Member of the Ministry of Health of the technical team).

Studies conducted within the scope of this sectoral policy highlighted the need for greater investments to achieve the ambition of the PNSPCD to ensure access to health services at the three levels of care (primary, secondary and tertiary) (Bernardes *et al.*, 2009; Tomaz *et al.*, 2016; Machado *et al.*, 2018). The empirical results highlight the advances made in the context of legislation. However, they also showed that this achievement was not accompanied by the guarantee of comprehensive health care for people with disabilities, given the insufficient funding of this policy.

Another important issue is that the services remained fragmented, as well as underfunded hindering the implementation of the PNCSPCD across the three levels of care (Machado *et al.*, 2018; Usine *et al.*, 2018). The interviewees also made a strong link between the fragmentation of the network of care and the lack of guarantee of comprehensive care for people with disabilities. The fragmentation of the care network is clear in the lack of consistency between the various public policies, the gaps between specialized services and primary care and the lack of effectiveness of the health system in meeting the needs of people with disabilities. Additionally, there was lack of coordination between the philanthropic services, already consolidated, and the new public services enabled as of 2012, with the former benefitting from greater political influence and stronger relationships with the families. The former minister interviewed highlighted that despite the advances of Living Without Limits policy, there remained a great challenge in the mediation between the philanthropic network and the public network of SUS.

Over these 11 years of progressive governments, advances were achieved both in the propositional content of the policy, as well as in the more democratic process of its formulation and implementation. However, there remained a gap between what was stated as policy guidelines and what was actually being implemented, especially with regard to the guarantee of comprehensive health care for people with disabilities. The expansion and integration of basic and specialized networks required to meet the specific healthcare needs of people with disabilities, as well as additional financial resources to ensure the financial sustainability of this sectoral policy remained a major challenge for the implementation of the policy.

### The Temer government (2015–18) and Bolsonaro (2019–present): a return to the past?

The chronic crisis of over-accumulation of capital and the economic recession made the Temer government choose to accelerate the fiscal adjustment, already started in the second Dilma government. In a more structural way, his government’s period is marked by the return of the liberalizing bias in the State, albeit with widespread concern as many people resisted losing the provisions and gains from the Lula and Dilma era. This period was characterized by the implementation of the fiscal austerity policy that transformed the chronic underfunding of the SUS into definancing, jeopardizing the sustainability of a system that purports to be universal, integral and equitable (Menezes *et al.*, 2019).

Examples of this shift includes the approval of Constitutional Amendment No. 95 (EC 95) in 2016, which froze public spending on fundamental social policies for 20 years, including health, education and social assistance, and the approval of the labour counter-reform in 2017. On the other hand, this government prioritized spending on interest and amortization of the debt around 39.7% of the Union’s General Budget, in addition to the 406 billion reais (approximately 82 billion dollars) in tax waivers, according to an estimate by the federal revenue (Souza and Soares, 2019; Corrêa and Loural, 2020). Budget cuts in activities related to ‘citizenship rights’ that reduced from R$2.4 billion in 2016 to R$1.6 billion in 2017, representing a 47% cut, this budget that includes, also, the policy of person with disabilities (David, 2017). Overall, counter-reforms and fiscal austerity have repercussions on the lives of workers, as shown by Castro’s analysis of some indicators (Castro, 2020). For instance, the unemployment rate in the population over 14 years of age rose from 6.8% in 2014 to 12% in 2018. Meanwhile, there was a loss of 2 million jobs in the formal sector (i.e. employment with a work card). The income of the poorest decreased; the proportion of the population with income less than a quarter of the minimum wage per capita rose from 9.6% in 2014 to 11.6% in 2018. Consequently, there was a growth in income inequality evidenced by the increase in the Gini Index of per capita household income from 0.526 in 2014 to 0.545 in 2018. In health, the infant mortality rate rose between 2015 and 2016, a fact that had not occurred in the last 20 years and has been linked to the underfunding of the SUS (Castro, 2020).

The Bolsonaro government, widely considered ultraliberal, continued the policies of fiscal restraint and reaffirmed the spending cap. It also continued the reduction of worker protection with the pension reform and the increase in budget cuts in social policies (Castro, 2020). Zigoni *et al* attempted to estimate the size of the financial cuts (Zigoni *et al.*, 2019). They showed that out of a total budget cut of approximately 31.04 billion reais (about 6 billion dollars) in June 2019, a third was taken from the policies of education, labour, social assistance, citizenship rights, public security, housing, sanitation and agrarian organization, among others. The largest cut was in education, which alone sustained a cut of 18% of the total budget, totalling 5.84 billion reais. In the area of citizenship rights, the budget was reduced by 27%, affecting policies related to the protection of minority rights, women, the indigenous population, ethnic minorities, migrants and people with disabilities.

The former health minister interviewed, suggested the application of EC/95 implied a loss for the SUS in 2019 of almost 10 billion reais (around 1.8 billion dollars). These cuts have occurred at a time when SUS was already experiencing issues due to geographical inequalities, chronic underfunding and suboptimal private sector–public sector collaboration (Castro *et al.*, 2019), all of which are likely to have been increased further as a result of the COVID-19 pandemic. Collectively, these cuts and pressures also had repercussions for the care network for people with disabilities. For instance, the representative of the current coordination of the policy believed that the cuts impacted on the implementation of the care network, both in the maintenance of the existing network and in its expansion. This scenario may still worsen, as estimates by Moretti et al. suggest that between 2020 and 2036 there would be a loss of more than R$2 trillion reais for the SUS (357 billion dollars) (Moretti *et al.*, 2020).

The Temer and Bolsonaro governments have not only cut spending but also have threatened the historic achievements of various social segments, such as the indigenous health subsystem (Pontes and Santos, 2020). They are also changing the propositional content of several other policies, including some fundamental to improving the health of people with disabilities. The National Primary Care Policy/PNAB (Table 2), for instance, has been weakened with the dismantling action of the Family Health Strategy. The changes to the Mental Health Policy threaten the achievements of the Psychiatric Reform in the country (Cruz *et al.*, 2020). Recently, the decree 10.502/2020 (Table 2), establishing the National Policy of Special Education contravenes the provisions of The Brazilian Inclusion Law by undermining laws and policies that guarantee that people with disabilities can study in Brazil’s mainstream educational system (Table 2). A draft legislative decree (PDL 437/2020) is currently being processed in the Senate to stop the effects of Decree 10.502. For Morosini, Fonseca and Baptista, these projected changes contribute to the strengthening of the mercantile logic in public policy (Morosini *et al.*, 2020).

One of the subjects interviewed suggested that the Bolsonaro government was dismantling policies protecting the rights of people with disabilities gained in the previous eras and heralded a return to a charitable model, led by philanthropic and religious organizations.

*[*...*] Look, if he (BOLSONARO) lets it in his head, we are going back to medieval Brazil, before the Republic. Who will take care of the disabled, will be philanthropy, whoever has access, the evangelicals neo-Pentecostals, or something like that*. (Professor and researcher at a Brazilian Public University. He/she was the Former National Health Council executive secretary).

Most of the subjects interviewed agreed that the Lula and Dilma governments provided a greater political opening for the participation of social movements, such as the disability movement in the discussion and implementation of public policies, including the PNSPC. In contrast, the Bolsonaro government is believed to break with this tradition. An evidence of this process is Decree No. 9.759/2019, which discontinues several councils and colleges of the federal public administration and establishes new guidelines and functions for these establishments, alongside the already described budget cuts.

*I have no doubt that we only had a voice because we were in a government that valued human rights. If it were now… The movement exerted pressure. […] Today there is an impossibility of dialogue with the management (BOLSONARO), we were no longer able to discuss or deliberate in a space that was ours, right? […], so, all this went backwards from that moment on, everything became more difficult […]. This shows a loss of power, of political space, do you understand? This is a serious problem! […] in practice this is not solving anything, they have no budget*. (Person with disability, who was a former government policy manager)

In the quote above, the interviewee clearly described the political process surrounding the implementation of the policy. Another added: *“Arouca (a great public health thinker) used to say: it is not enough to be written, it needs to be permanently battled” (Member of the National Health Council).* Social movements influenced the establishment of the PNSPCD on the government agenda and continue over the years as key actors who exert strong pressure for the implementation of the policy. But their capacity to influence implementation and government policy fluctuated over time.

 Since the year of PNSPCD’s promulgation in 2002, a set of initiatives and strategies within this sectoral policy converged, even if insufficiently, for its expansion and strengthening. However, these advances have been threatened, given the conservative forces of the Bolsonaro government, guided by ultraliberal ideology, which have imposed successive setbacks in the field of social rights. Among these setbacks is the weakening of the social participation spaces and the slow-down in expansion of the SUS, and consequently PNSPCD’s de-funding process. This move away from a commitment to inclusion marks a return to a focus on care provision through philanthropic institutions to the detriment of strengthening of the public services.

 It is also worth noting that the Covid-19 pandemic had a negative impact on the guarantee of access to health services, although there were some safeguards put in place, they were much more tokenistic than actually effective. The Covid-19 pandemic hit the Brazilian population hard, and by August 2021 it had already claimed more than 560 000 lives. Without a doubt, the population of people with disabilities, due to its great vulnerability, was hit hard, which requires further studies to analyse in detail (Reichenberger *et al.*, 2020; Sakellariou *et al.*, 2020).

### Final considerations

 The analysis presented in this article suggest that the policy developments aimed at establishing and fulfilling the rights of people with disabilities to healthcare were born within the scope of philanthropy and were developed by specific and fragmented actions, although mostly supported from public funds. Different actors have played important roles over the last 50 years, including the disability movement, technical staff, philanthropic institutions, international organizations and governments of different political and ideological shades. The political contexts in which these developments occurred varied, at times favourable to the inclusion of their demands in the government agenda, sometimes with setbacks during the process of its implementation. Undoubtedly, the neoliberal offensive on social policies, especially the SUS, is the main obstacle to the consolidation of the PNPCD. The policy was formulated in the FHC governments, constituting a breakthrough for the Rights of persons with disabilities by strong coordination of the technical staff of the Ministry of Health with agendas and demands of social movements. However, this articulation was not sufficient for the implementation of that policy.

 The research suggests that the Lula and Dilma governments were marked by significant advances for the right to healthcare of people with disabilities, although with contradictions. During the Lula government, Brazil became a signatory to the Convention on the Rights of Persons with Disabilities, which opened a window of opportunity for social movements to influence the incorporation of their demands and the principles of the convention into the Brazilian regulatory framework. However, this alignment with the Rights of Persons with Disabilities did not translate into the implementation of a comprehensive health care network. The era of President Dilma saw the creation of an intersectoral plan called Living without Limitation, under which more specific strategies were developed to implement health care network for people with disabilities and for the first time with specific funding lines. However, the PNPCD, and the SUS more broadly, remained underfunded despite the adoption by these centre-left governments of important economic and social policies that were able to foster economic growth in the country, eradicate poverty and provide improvements in a set of socio-sanitary indicators. As a practical consequence, there was a mismatch between the proposals in the policy guidelines and what was actually being executed.

 From 2017, SUS and consequently the PNPCD experienced defunding, in the face of the ultraliberal government and expansion of the counter-reform project of the State, especially with regard to social policies. In addition to financial issues, the current Bolsonaro government has brought significant setbacks in the field of social participation, emptying the spaces of political struggle and from a government agenda of authoritarian and conservative principles, it also diminished its propositional contents. Thus, the research suggests that the policy returns to having a welfare and rehabilitative character, with a focus on delivery of services by philanthropic institutions to the detriment of the formation of a policy of comprehensive health care to the person with disabilities.

 There are strengths and limitations to the study, and the use of the Walt and Gilson framework, which must be taken into account when interpreting the findings. The case study was ambitious in scope, using a multidisciplinary approach and both describing what happened in the development of PNSPCD and attempting to explain why events unfolded in this way over almost three decades. The high ambition of the study and the broad scope of the Walt and Gilson framework meant that certain aspects could not be addressed in detail. For instance, actors considered were not comprehensive (e.g. lack of focus on cross-border actors), and there was a relatively limited focus on the content and implementation of the policies. Indeed, an analysis could have been possible of each component of the framework alone, or taking a different perspective such as the role of social movements in framing the policy development (Benford and Snow, 2000). However, we believe there was value in looking at the holistic picture and how the components interacted through the Walt and Gilson framework. We attempted to explain why the PNSCPD developed along certain trajectories but acknowledge that ‘Decision making processes are often opaque’, (Walt *et al.*, 2008) particularly to researchers outside of the policy making sphere attempting to piece together the narrative many years after the event. Moreover, in this retrospective analysis interviewees reports on what happened at the time may be influenced by subsequent events, creating the possibility of recall bias. The findings may also have been influenced by our own position. The research team predominantly consisted of Brazilian researchers who were knowledgeable about the context. However, our interpretation could have been influenced by our position outside of the policy development space, our left-wing political perspective and our belief in the importance of disability rights. Another concern is that this analysis presents one case study of one policy formulation in one setting. Further comparative case studies would be helpful to improve generalizability of the findings.

In conclusion, the research findings demonstrate that the development and implementation of public policies experiences advances and retreats, determined, above all, by national and international macro-political decisions. The prominent feature of the current era is fiscal austerity and dismantling of social policies. Campaigning for specific laws on the rights of access to health is a start, but is not sufficient, to ensure equitable healthcare for people with disabilities. The wider social context and the state of the health system is essential. As such, the sustainability of the SUS likely requires the repeal of EC/95, in addition to the expansion and restructuring of investments, given the ageing of the population, the growing inequalities in Brazil and the ongoing impacts of the COVID-19 pandemic. Furthermore investment, both in amount and focus, is needed in order to achieve the goals of the PNSPCD and to comply with the Brazilian legal protections for access to healthcare of persons with disabilities. Strengthening of spaces for social participation in the implementation of this sectoral policy is likely to be a helpful step in this process.

## Reflexivity statement

This paper has been written by a team of researchers based in Brazil and London who have a long-established relationship and have carried out research and published together before. We do not all have one language in common. The vast majority of data extraction and analysis were completed by Brazilian-based authors.  We have spent a great deal of time thinking through and discussing the various contributions that we bring to the paper and the arguments and approaches used in the paper.  The Brazilian team was very keen to publish in an English language international journal. The second to last and last authors have experience publishing in English.  Through our collaboration, we have worked hard to work in both Portuguese and English thinking very carefully about the language of different drafts to allow full participation of all team members. There is always, however, the danger that some things are lost in translation. In this project, as with the previous project, we have also experienced the challenge that not only do we speak different languages but also have we often read different literatures and are used to writing in different styles. The English authors are interested outsiders while the Brazilian team members are highly engaged and deeply knowledgeable about the Brazilian health system and social movements.  We hope that our different experiences and approaches complement each other—but we also recognize that in a relatively short article we have simplified what is a complicated policy process.
